# Overcoming limitations in the availability of swabs systems used for SARS-CoV-2 laboratory diagnostics

**DOI:** 10.1038/s41598-021-81782-8

**Published:** 2021-01-26

**Authors:** Manfred Nairz, Rosa Bellmann-Weiler, Miriam Ladstätter, Falko Schüllner, Martina Zimmermann, Anna-Maria Koller, Silvia Blunder, Helene Naschberger, Werner Klotz, Manfred Herold, Sylvia Kerndler, Martina Jeske, David Haschka, Verena Petzer, Andrea Schroll, Thomas Sonnweber, Ivan Tancevski, Gernot Fritsche, Mariana E. G. de Araujo, Taras Stasyk, Lukas A. Huber, Andrea Griesmacher, Igor Theurl, Günter Weiss

**Affiliations:** 1 Department of Internal Medicine II, Infectious Diseases, Immunology, Rheumatology, Pneumology, Medical University of Innsbruck, Anichstr. 35, 6020 Innsbruck, Austria; 2 Hospital Pharmacy, University Hospital of Innsbruck, Innsbruck, Austria; 3 Department of Internal Medicine V, Hematology and Oncology, Medical University of Innsbruck, Innsbruck, Austria; 4 Institute of Cell Biology, Biocenter, Medical University of Innsbruck, Innsbruck, Austria; 5 Central Institute of Medical and Chemical Laboratory Diagnostics, University Hospital of Innsbruck, Innsbruck, Austria

**Keywords:** Infectious-disease diagnostics, Viral infection

## Abstract

The diagnosis of COVID-19 relies on the direct detection of SARS-CoV-2 RNA in respiratory specimens by RT-PCR. The pandemic spread of the disease caused an imbalance between demand and supply of materials and reagents needed for diagnostic purposes including swab sets. In a comparative effectiveness study, we conducted serial follow-up swabs in hospitalized laboratory-confirmed COVID-19 patients. We assessed the diagnostic performance of an *in-house* system developed according to recommendations by the US CDC. In a total of 96 serial swabs, we found significant differences in the accuracy of the different swab systems to generate a positive result in SARS-CoV-2 RT-PCR, ranging from around 50 to 80%. Of note, an *in-house* swab system was superior to most commercially available sets as reflected by significantly lower Ct values of viral genes. Thus, a simple combination of broadly available materials may enable diagnostic laboratories to bypass global limitations in the supply of swab sets.

## Introduction

Coronavirus disease 2019 (COVID-19) is the respiratory tract infection caused by severe respiratory distress syndrome coronavirus 2 (SARS-CoV-2)^1–3^. The diagnosis of COVID-19 largely relies on the direct detection of viral ribonucleic acid (RNA) in respiratory specimens^4,5^. In most cases, specimens are obtained from the upper respiratory tract by swabbing the posterior nasopharyngeal or oropharyngeal wall using commercially available sets^6,7^. These sets typically consist of a plastic swab and a tube containing universal or virus transport medium (UTM or VTM, respectively) in compositions and quantities that differ between the manufacturers.

Once collected, respiratory specimens are most commonly analyzed in diagnostic laboratories by reverse transcription polymerase chain reaction (RT-PCR) to detect viral RNA^8,9^. Some RT-PCR tests are developed *in-house*, often following recommendations of reference laboratories and official institutions^10–12^. Other tests are commercially available and are in research use only (RUO) status or certified as in-vitro diagnostics (IVD)^13–16^. Typically, RT-PCR tests are run in multiplex to detect two viral genes and one internal control in the same reaction well, tube or cartridge.

In many regions hit by the COVID-19 pandemic, health authorities have put containment strategies in place^17^. Among these, broad RT-PCR testing of asymptomatic or symptomatic individuals, restrictions in transportation, tracing of social contacts, barrier precautions, isolation and quarantine measures are the most common ones^18–21^. While the detection of SARS-CoV-2 by RT-PCR is highly specific for COVID-19, the sensitivity of the method is limited by several factors, including the employed swab sets^22–24^. Therefore, in suspected cases, clinical, biochemical and radiological findings are important in confirming the diagnosis of COVID-19^25–27^.

Broad screening by RT-PCR requires accurate planning and concerted use of the material and personnel resources for the proper collection, administration and actual molecular analytics of specimens^5,28–30^. On a large scale, these steps and the subsequent interpretation and submission of results challenge the ordering, warehousing and other infrastructural aspects of diagnostic laboratories^31^. Therefore, large regional and national screening programs require enormous effort in preanalytical, analytical and postanalytical phases of laboratory diagnostics.

Because of the unprecedentedly high number and urgency of tests requested, diagnostic laboratories in endemic regions including ours faced the challenge to acquire sufficient amounts of materials and reagents. Despite established supply chains, the most striking shortages occurred in specimen collection sets and RT-PCR reagents^32^.

As for RT-PCR, several national reference laboratories developed and published primers, probes and test conditions^10,15^. With a little delay, many manufacturers entered the growing market with commercially available kits whose status ranged from RUO products to *in-vitro* diagnostics with European CE marking (CE-IVD). Due to the prompt response of the biomedical industry and the growing availability of test kits for RT-PCR, limitations moved towards more general materials and reagents such as swabs and chemicals for RNA extraction. In our study, we systematically validated commercially available swab sets as well as a system produced *in-house* for their practical utility in collecting oropharyngeal swabs and their biochemical compatibility with the subsequent diagnostic RT-PCR for SARS-CoV-2.

## Results

### The positivity rate of follow-up SARS-CoV-2 RT-PCR results from confirmed cases varies between swabs systems

We enrolled symptomatic patients hospitalized for acute laboratory-confirmed COVID-19 at the Department of Internal Medicine at the University Hospital of Innsbruck, Austria. Oropharyngeal swabs were taken for follow-up within the first five days after admission to the hospital. The mean time interval between the initial swab with positive RT-PCR result and the follow-up sampling was three days, and there were no apparent differences between these intervals across the swab systems tested (Supplemental Fig. S1). Subsequently, the diagnostic effectiveness of different systems used for follow-up swabs that were serially taken on one occasion (within 1–5 min) was retrospectively analyzed.

We compared the utility of different swab systems in confirming the initial positive RT-PCR result that led to the classification of the case as laboratory-confirmed COVID-19. We saw a wide range in the percentage of results that were repetitively positive using the same RT-PCR method within five days of the initial test. Specifically, the percentages ranged between 50.0% and 81.3%. Interestingly, none of the collection systems could confirm the initially positive RT-PCR result to 100% and only three swabs sets reached an accuracy in confirming the initial results with 70% or more.

**Figure 1:**
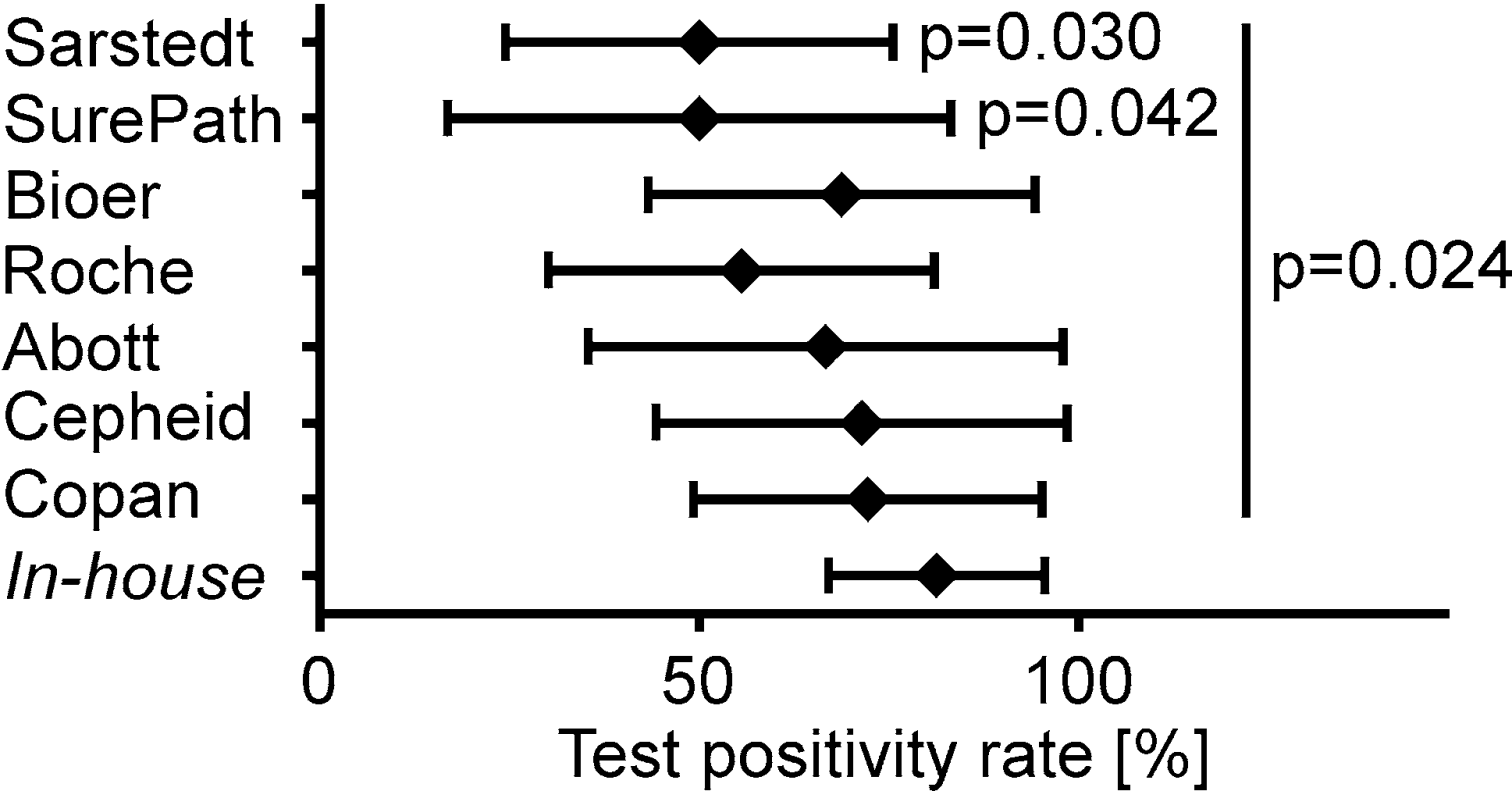
The positivity rate of SARS-CoV-2 RT-PCR as a function of the swab system used. Oropharyngeal samples from patients hospitalized for COVID-19 were taken with different swab sets. The percentage of samples re-tested positive for SARS-CoV-2 is depicted as mean ± 95% confidence interval. n = 32 for the *in-house* system, n = 18 for the Copan system, n = 12 for the Cepheid system, n = 12 for the Abott system, n = 18 for the Roche system, n = 16 for the Bioer system, n = 12 for the BD SurePath system, n = 18 for the Sarstedt swab. p = 0.042 for *in-house* vs. SurePath. p = 0.030 for *in-house* vs. Sarstedt. p = 0.024 for *in-house* vs. all other systems combined.

Initially, we had two swab sets available because they were already validated for use in the laboratory diagnosis of influenza by RT-PCR: The swab set with UTM obtained from Copan reached a mean sensitivity of 70.0% (95% CI of mean 48.0–92.0%) in the SARS-CoV-2 RT-PCR. The swab set with VTM from Cepheid reached 71.4% (95% CI of mean 44.4–98.5%). For comparison, the *in-house* system containing VTM mixed according to a CDC recipe reached 81.3% sensitivity (95% CI of mean 67.0–95.6%) (Fig. 1). The re-confirmation of the initial positive PCR result was much lower when using Sarstedt dry swabs followed by elution with an isotonic saline solution or the BD SurePath system, intended for cervical swabs in liquid-based gynecologic cytopathology and widely used for Human Papilloma Virus (HPV) diagnostics^33^. The latter two systems produced only 50.0% positive results (95% CI of mean 22.5–77.5% for the SurePath system and 26.0–74.0% for the dry swab method, respectively).

### The analytical sensitivity of SARS-CoV-2 RT-PCR is dependent on the swab system used for specimen collection

**Figure 2:**
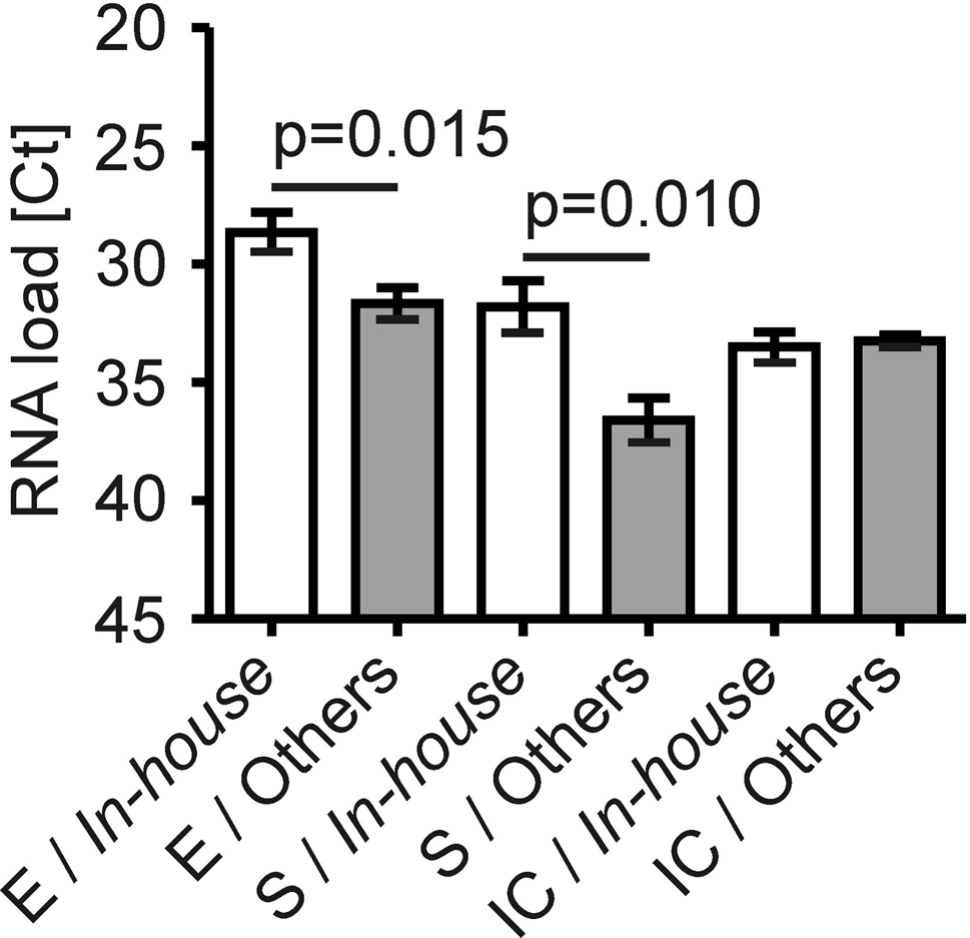
The sensitivity of SARS-CoV-2 RT-PCR depends on the swab system used. Ct values of all positive RT-PCR results taken with either of eight swabs variants were separated in two categories, i.e. the *in-house* system (white bars) and all other systems combined (grey bars). n = 26 for the *in-house* system, n = 70 for all other variants. Data are depicted as mean ± SEM for the viral E gene, the viral S gene and the internal control (IC). Statistically significant differences are indicated.

To further substantiate these findings, we looked into Ct threshold cycle (Ct) values of our RT-PCR results. Although the Altona RealStar SARS-CoV-2 RT-PCR is a qualitative RT-PCR by design, the Ct values are proportional to the amount of RNA present in the sample. We thus analyzed the Ct values for the SARS-CoV-2 envelope (SARS-CoV-2 E) gene, for the SARS-CoV-2 spike (SARS-CoV-2 S) gene and for the internal control (IC) of all 96 positive follow-up samples. When we compared the SARS-CoV-2 positive samples collected with the *in-house* system to all other systems combined, we found that the Ct values of the viral E gene and viral S gene were significantly lower for the *in-house* system (Fig. 2; inverse y-axis). In contrast, the Ct values of the initial RT-PCR from the first diagnostic swab (all taken with the Cepheid system) were comparable between the *in-house* swab system and all other systems (Supplemental Fig. S2; inverse y-axis). Taken together, these results were again indicative of high analytical sensitivity of the *in-house* system as compared to commercially available swab sets.

In detail, we found that the Ct values for the E gene obtained with the *in-house* swab set were the lowest, amounting to 28.65 (95% CI of mean 26.92–30.38), which indicated the highest analytical sensitivity (Supplemental Fig. S3A; inverse y-axis). The Ct values for the E gene obtained with the mentioned Cepheid swab system were the second-lowest, amounting to 29.45 (95% CI of mean 24.04–34.86). When assessing the S gene, we saw that the Ct values with the *in-house* system were the second-lowest, amounting to 31.80 (95% CI of mean 29.55–34.06) (Supplemental Fig. S3B; inverse y-axis). Notably, the Ct values for the IC were very similar across all swab sets tested (Supplemental Fig. S3C; inverse y-axis).

**Figure 3:**
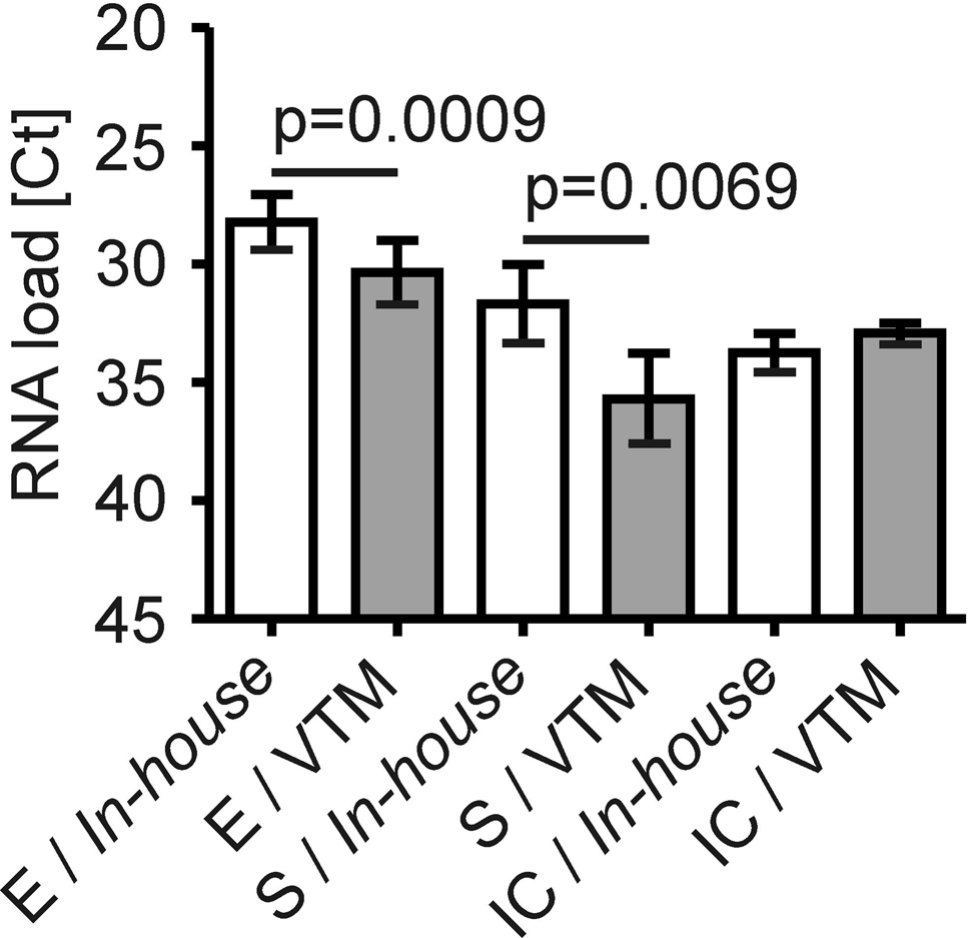
Direct comparison of the *in-house* swab system with two previously validated collection sets. Ct values of paired positive RT-PCR results taken with the *in-house* system (white bars) and either of two swabs sets validated for pharyngeal swabs (i.e. the Copan and Cepheid VTM sets; grey bars). n = 19 paired samples. p = 0.0009 for the Ct values of the viral E gene between the two groups.

In 19 additional pairwise swabs, we had Ct values of direct comparisons between the *in-house* system and either of our two sets originally validated for pharyngeal swabs, from Copan or Cepheid, available. Strikingly, we observed significantly lower Ct values for the E gene but not the S gene and the IC using the *in-house* system (Fig. 3; inverse y-axis). This indicated that the *in-house* swab system indeed formed a suitable basis for highly sensitive detection of SARS-CoV-2 RNA by RT-PCR.

### The volume of virus transport medium used for specimen collection has a minor effect on the diagnostic performance of SARS-CoV-2 RT-PCR

Next, we asked whether the volumes of the liquid media provided may affect the sensitivity of the swab system for subsequent SARS-CoV-2 RT-PCR. We found that the volume of the VTM present in the *in-house* system had a minor effect on the Ct values for the viral E gene which were very similar for sets with 1.2 ml, 2 ml, 3 ml and 4.3 ml of VTM (Supplemental Fig. S4A; inverse y-axis). The same was true for commercially available swab systems which use different volumes of liquid media according to the manufacturers’ specifications. A very similar pattern of Ct values was observed for the viral S gene (Supplemental Fig. S4B; inverse y-axis), while for the IC, Ct values were essentially the same across the swabs systems compared (Supplemental Fig. S4C; inverse y-axis).

### Organic compounds in swabs may inhibit SARS-CoV-2 RT-PCR

Since back orders not only affected tubes with VTM but also plastic swabs, we combined the *in-house* tube containing 1.2 ml of VTM with either of six different swabs. We used the Puritan PurFlock Ultra Flocked Swab (Pure), the Copan FLOQSwabs Copan Flocked Swabs (Flocked), the Copan CLASSIQSwabs (Classic), the mwe medical wire DRYSWAB (Dry), a clean room product intended for the cleaning of technical devices termed IAB Reinraumprodukte GmbH Polyester Swab PS3605 (Technical) and the wooden Raucotupf Cotton tipped Applicator (Wooden). In serial swabs from four individual patients, we found that the viral E gene was detectable with comparable Ct values in all samples but not in that one taken with the wooden cotton-tipped swab (Supplemental Fig. S5A; inverse y-axis). The viral S gene was detectable in three out of four samples taken with most swabs. However, the viral S gene was undetectable in two out of four samples taken with the technical swab and was undetectable in all samples taken with the wooden cotton-tipped swab (Supplemental Fig. S5B; inverse y-axis).

**Figure 4:**
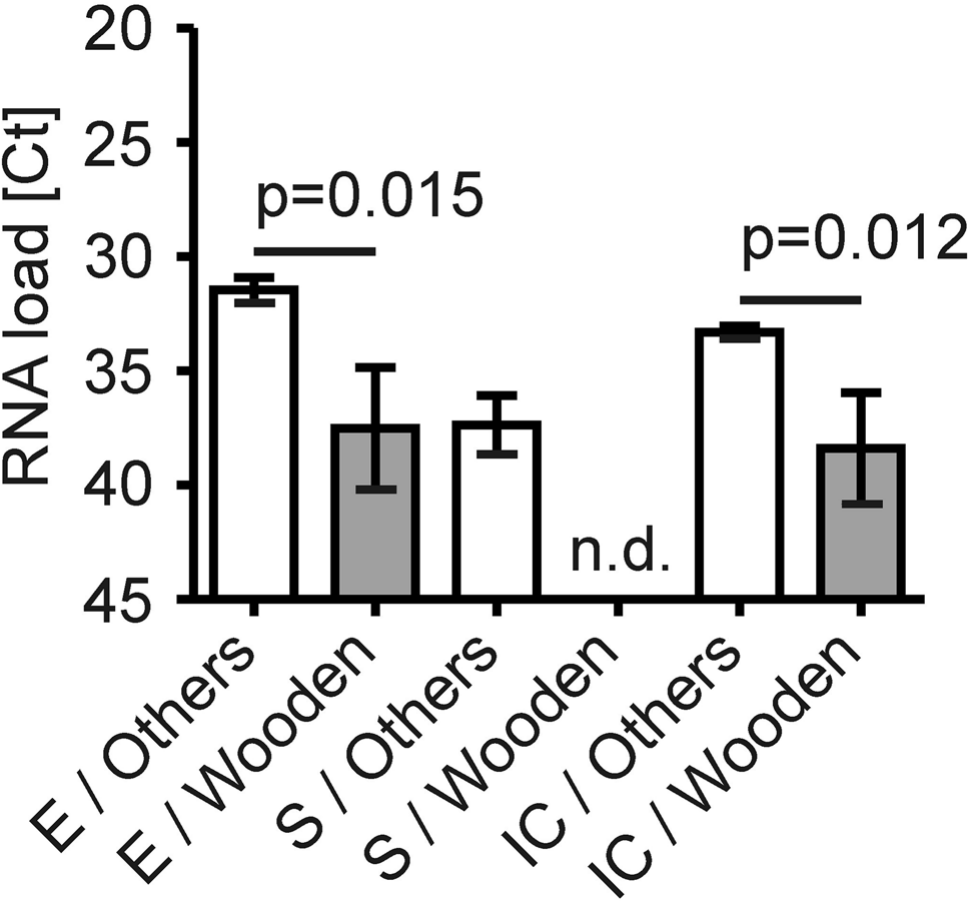
Organic compounds affect the sensitivity of SARS-CoV-2 RT-PCR. Oropharyngeal samples taken with other swabs (white bars) were compared to those taken with wooden swabs from Raucotupf (grey bars). Ct values of the RT-PCR results presented in Supplemental Fig. S5 were compared between these two categories. n = 20 for the other system, n = 4 for the wooden variant from Raucotupf. Data are depicted as mean ± SEM for the viral E gene, the viral S gene and the internal control (IC). Statistically significant differences are indicated. n.d. for not detected.

The IC was similar across all swab systems but lower, albeit not significantly, in the group taken with the wooden cotton-tipped swab in which one IC remained undetectable, resulting in a single invalid test result (Supplemental Fig. S5C; inverse y-axis). Therefore, we next compared the wooden cotton-tipped swab with all other variants of swabs and saw that its sensitivity as deduced from the higher Ct values of RT-PCR results was significantly lower (Fig. 4; inverse y-axis).

### The virus transport medium stabilizes SARS-CoV-2 RNA during storage in the refrigerator

**Figure 5:**
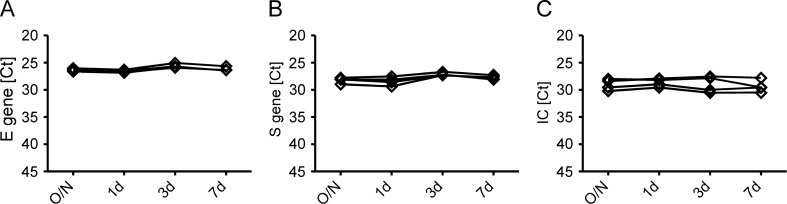
The stability of SARS-CoV-2 RNA in the *in-house* VTM. The stability of the *in-house* VTM was tested by separating 4 ml samples taken from four SARS-CoV-2 positive patients into four aliquots. These aliquots were stored in the refrigerator at 2–8 °C overnight (O/N), for 24 h (1d), for 72 h (3d) or 168 h (7d). Thereafter, nucleic acids were extracted for RT-PCR. Data are depicted as individual Ct values for the viral E gene (**A**), the viral S gene (**B**) and the internal control (**C**). No statistically significant differences between the time points were found.

Next, we tested the ability of the *in-house* swabs system to preserve viral RNA for detection by RT-PCR. To this end, we measured aliquots of samples after storage at 4 °C overnight (O/N), for 24 h (1d), 72 h (3d) or 168 h (7d) by RT-PCR. We saw that the Ct values of the viral E gene, viral S gene and the IC remained fairly stable during the observation period (Fig. 5A–C; inverse y-axis). The addition of 4000U/ml of an RNAse inhibitor for 72 h had no substantial effect on Ct values of the E gene in specimens collected with the *in-house* (white bars) or Copan system (grey bars) used as original reference (Supplemental Fig. S6; inverse y-axis). These results also indicate that the sterile Sarstedt tubes, used for the *in-house* system that have up to now not been reported to be free of RNases, could be used without the addition of an RNase inhibitor.

## Discussion

RT-PCRs for SARS-CoV-2 are performed for various indications. First, asymptomatic individuals may be screened because they had previously been in close contact with somebody diagnosed with COVID-19. This approach is considered a public health measure, because it detects SARS-CoV-2 infection early in pre-symptomatic subjects and enables the breach of infection chains^13,34,35^. Second, asymptomatic health-care workers and other staff working in hospitals or nursing homes, who are in contact with vulnerable groups of patients, can be screened for the safety of both co-workers and patients^36,37^. Third, individuals with symptoms suggestive of COVID-19 such as fever, malaise, dry cough or loss of taste or smell ought to be tested for diagnostic purposes and initiation of subsequent specific health care measures.

Given these diverse test indications and the pandemic spread of SARS-CoV-2, PCR testing needs to be conducted on a large scale and with high sensitivity not only in a given country but worldwide. As a consequence, millions of swabs and PCR kits are needed globally during the ongoing pandemic, challenging resources and supply chains.

Confronted with limitations in resources and logistics, we investigated the utility of several kinds of swab sets in a well-defined cohort of subjects. We recruited 64 symptomatic patients who were confirmed to have COVID-19 by RT-PCR and who were hospitalized for supportive care such as conventional oxygen therapy. These patients were re-swabbed for follow-up within five days (mean approximately 3 days; range 1–5 days) after the initial positive RT-PCR. The focus of our study was the technical validation of alternative swabs systems used to collect oropharyngeal specimens and their sensitivity in identifying PCR-confirmed cases. However, one has to admit that PCR results may turn negative due to virus clearance after a short period of time^38^.

The earliest and simplest way to approach limitations in swab sets was to use dry plastic swabs which are compatible with PCR as recommended by some authorities. However, collecting an upper respiratory sample with a dry swab and eluting the viral RNA contained in the specimen with normal saline may reduce the diagnostic sensitivity of the subsequent RNA extraction and RT-PCR, consequently^39,40^. This limitation has been known for other pathogenic viruses and was confirmed for SARS-CoV-2 in our study (Fig. 1). Therefore, we aimed to improve the yield of viral RNA by mixing a VTM *in-house* from a small number of components that are more broadly available because many vendors deal with those reagents. However, we also purchased other specimen collection sets and included them in our comparative analysis. For instance, sets used in gynecology or urology which are suitable for the detection of *Chlamydia*, *Mycoplasma* and *Ureaplasma* spp. but also HPV, were evaluated to collect specimen from the posterior oropharyngeal wall. Furthermore, the *in-house* VTM was used in combination with several swabs. However, when using one with a wooden stalk and a cotton tip, we failed to detect SARS-CoV-2 by RT-PCR. Concretely, we saw that the Raucotupf swab resulted in high Ct values for the viral E gene and undetectable levels of the viral S gene as surrogates of poor sensitivity. Organic compounds present in wood and cotton could have inhibited PCR reactions. Specifically, tannic acid present in wood has been demonstrated to inhibit Taq polymerase^41^.

Another option to circumvent backorders in plastic swabs is to produce them in a 3D printer as recently validated in other laboratories^42,43^. However, plastic ware for molecular biology applications may require particular specifications and purities. This may be especially relevant for RNases that can contaminate surfaces including the outside of swabs or the inside of tubes or be present in biological samples^44^. However, in our validation, we did not see any effect of the addition of an RNase inhibitor that would prevent the negative effects of possible contamination of collection tubes. Thus, when tubes are produced under clean room conditions and handled with gloves during sampling and laboratory work-up, RNases may not be of concern for detecting SARS-CoV-2 by RT-PCR.

Our study has several limitations. First, the study was monocentric by design, precluding general recommendations. Second, we focused on the Altona Realstar RT-PCR system in RUO status, which detects the E gene and the S gene along with an internal extraction and amplification control. However, when we validated its diagnostic performance in comparison with the Roche Cobas SARS-CoV-2 test or an *in-house* RT-PCR for SARS-CoV-2 using the primers and probes published by the CDC, we found no significant differences between these methods, suggesting that our results may not depend on a specific target sequence or type of polymerase. Third, we only enrolled hospitalized patients with moderate symptoms, who may have been in the disease state of ongoing viral clearance. Fourth, our study exclusively deals with oropharyngeal swabs, a common specimen taken for SARS-CoV-2 PCR^45^ because we had observed in advance that serial sampling at the posterior oropharyngeal wall is technically less challenging for health-care professionals, physically less demanding for test subjects and thus more reproducible than in the nasopharyngeal cavity. Fifth, due to a limited number of eligible patients (n = 64), the sample size of our study is rather small (n = 96 serial swabs and n = 19 pairwise swabs) for assessing a pandemic disease and further studies which have a prospective and multicentric design or recruit out-patients as well may be required for more general conclusions.

One of the most striking findings of our study is that none of the swabs systems used could confirm the previous RT-PCR diagnosis of COVID-19 with 100% accuracy. In fact, the *in-house* system reached the highest percentage of *bona fide* true positive results, concretely, 81.3%. While these numbers may first appear counterintuitively low, they support the idea that the timing and mode of specimen collection, transport and storage conditions as well as the sensitivity of the subsequent RT-PCR are critical for an accurate diagnosis. Therefore, when the clinical suspicion for infection is high, repeated testing may be required which will consume additional resources, though^46,47^. Furthermore, many reports suggest that some patients with COVID-19 can display intermittently or continuously negative RT-PCR results in upper respiratory specimens^48,49^. In addition, the host response directed against SARS-CoV-2 may either reduce the virus load below the detection limit of our RT-PCR (0.1 PFU/ml as reported by the manufacturer) or completely clear the virus.

In summary, our study emphasizes that materials and methods have to be thoroughly validated in diagnostic laboratories. This is especially relevant when alternative systems are adapted^50^. We could also demonstrate that, by employing published protocols, the cooperation of the diagnostic laboratories and the Hospital Pharmacy at a tertiary center was able to circumvent limitations in the regional supply of swab sets from the global market. Our results argue for stringent standardization of specimen collection not only for diagnostic SARS-CoV-2 PCR but also for intervention studies on COVID-19 many of which include RT-PCR results as secondary end points.

## Materials and methods

The swab/media sets tested were as follows (1) the *in-house* system, consisting of 1.2 ml of VTM (for virus transport medium), prepared according to CDC recommendations and filled into a 4.5 ml Sarstedt polypropylene tube, (2) the Copan UTM (order number 305C), containing 3 ml of UTM (for universal transport medium), (3) the Cepheid GeneXpert Xpert Nasopharyngeal Sample Collection Kit for Viruses, containing 3 ml of VTM, (4) the Abott multi-Collect Specimen Collection Kit, containing 1.2 ml media, (5) the Roche cobas Dual Swab Sample Kit, containing 4.3 ml PCR Media (6) the Bioer Nasopharyngeal Swab, containing 2 ml of Sample Preservation Fluid, (7) a neutral Sarstedt Rayon viscose swab (order number 80.1301), used in combination with a 4.5 ml Sarstedt polypropylene tube and 1 ml of normal saline and (8) the BD SurePath containing 10 ml Preservative Fluid.

The *in-house* system was prepared in the Innsbruck Hospital Pharmacy and validated in the Infectious Diseases (ID) laboratory according to the Austrian Medical Devices Act. To this end, 1.2 ml of VTM were filled into a 4.5 ml Sarstedt polypropylene tube (order number 60.557.001). The mixture of the VTM was published by the CDC. It was based on Hanks Balanced Salt Solution (1xHBSS) with calcium and magnesium ions, without phenol red, and contained 2% of heat-inactivated fetal bovine serum (FBS), 100 μg/ml gentamicin and 0.5 μg/ml amphotericin B. The components were mixed under aseptic conditions in a laminar air flow unit and remained sterile as confirmed by microbiologic testing. Moreover, the *in-house* system could be stored at 2–8 °C prior to use for at least 8 weeks without loss of sensitivity in the RT-PCR for SARS-CoV-2 (details not shown).

The swabs used in combination with the *in-house* system were as follows (1) the Puritan PurFlock Ultra Flocked Swab, (2) the Copan FLOQSwabs Copan Flocked Swab, (3) the Copan CLASSIQSwab, (4) the MWE medical wire DRYSWAB, (5) the clean room product IAB Reinraumprodukte GmbH Polyester Swab (order number PS3605) and (6) the Lohmann&Rauscher Raucotupf Cotton tipped Applicators (order number DE-01369204).

Two biomedical technicians performed RNA extraction and RT-PCR in the infectious diseases diagnostic laboratory using a robotic pipetting workstation: We used 500 µl of the respective VTM, the AltoStar Purification Kit 1.5 and the RealStar SARS-CoV-2 RT-PCR Kit 1.0 RUO (order number 821005) ran on the AltoStar Automation System AM16. The detection limit of the RT-PCR reported by the manufacturer is 0.1 plaque-forming units/ml. RT-PCR was run for 45 cycles on a C1000 Touch Thermal Cycler manufactured by Bio-Rad Laboratories and provided by Altona. The RT-PCR result of a given sample was considered positive for SARS-CoV-2 when either the E gene or the S gene or both viral genes were detectable. When neither one of the viral genes nor the internal control (IC) were detectable, the RT-PCR was considered invalid. Oropharyngeal samples were kept on room temperature from specimen collection to acceptance in the ID laboratory. Unless otherwise specified, samples were then stored overnight in the refrigerator at 2–8 °C. Thereafter, the plastic swab was removed and the collection tube containing transport medium or preservation fluid was kept on room temperature until nucleic acid extraction. Nucleic acid extraction and RT-PCR were performed in a closed, CE-IVD approved system.

### Ethics

Oropharyngeal swabs were collected for follow up of COVID-19 patients who were treated on wards dedicated to normal care in ID at the Department of Internal Medicine, University Hospital of Innsbruck, and for technical validation of the systems in the ID laboratory. Written informed consent was obtained from all participants and/or their legal guardians for the collection, processing and retrospective comparative analysis of the swab systems. The study was approved by the local ethics committee of the Medical University of Innsbruck (‘Ethikkommission der Medizinischen Universität Innsbruck’, approval numbers 1091/2020, 1103/2020 and 1167/2020), and all research was performed in accordance with the relevant guidelines and regulations.

### Statistics

Statistical analysis was performed with the GraphPad Prism 5 software package. Data distribution and homoscedasticity were analyzed. Accordingly, we used Fisher's exact test to analyze categorical variables and Mann–Whitney U test, Kruskal–Wallis or Wilcoxon test to analyze continuous data. Dunn correction was applied to adjust alpha error for multiple testing.

## Supplementary information

Supplementary Information

## Data Availability

The datasets generated during and/or analysed during the current study are available from the corresponding author on reasonable request.
