# Supplementary material for: Overcoming limitations in the availability of swabs systems used for SARS-CoV-2 laboratory diagnostics
Source: Sci Rep. 2021 Jan 26;11:2261. doi: 10.1038/s41598-021-81782-8 (PMC7838421; doi:10.1038/s41598-021-81782-8)
Supplement: Supplementary file 1 — Supplementary Information [file 41598_2021_81782_MOESM1_ESM.pdf]

Overcoming Limitations in the Availability of Swabs Systems Used for SARS-CoV-2 Laboratory Diagnostics

Manfred Nairz[1,*], Rosa Bellmann-Weiler[1,#], Miriam Ladstätter[2,#], Falko Schüllner[2,#], Martina Zimmermann[1], Anna-Maria Koller[1], Silvia Blunder[1], Helene Naschberger[1], Werner Klotz[1], Manfred Herold[1], Sylvia Kerndler[2], Martina Jeske[2], David Haschka[1], Verena Petzer[3], Andrea Schroll[1], Thomas Sonnweber[1], Ivan Tancevski[1], Gernot Fritsche[1], Mariana E. G. de Araujo[4], Taras Stasyk[4], Lukas A. Huber[4], Andrea Griesmacher[5], Igor Theurl[1], Günter Weiss[1]

Supplemental Figures and Supplemental Figure Legends.

Supplemental Figure 1

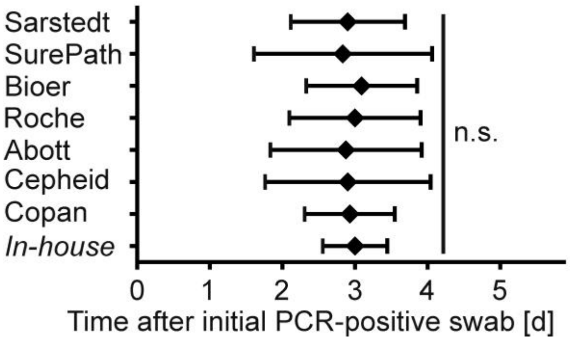

Supplemental Figure 1. The time interval between the initial swab and the follow-up sampling is approximately three days and comparable between the systems tested.

The mean time interval between the initial swab with positive PCR result and the follow-up sampling was retrospectively analyzed. n=26 for the *in-house* system, n=14 for the Copan

system, n=10 for the Cepheid system, n=8 for the Abott system, n=11 for the Roche system, n=11 for the Bioer system, n=6 for the BD SurePath system n=10 for the Sarstedt swab. No significant difference in the time interval was found across swab types, confirming appropriate randomization. n.s. for not significant.

Supplemental Figure 2

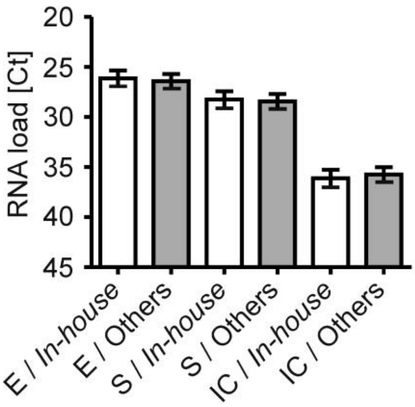

Supplemental Figure 2. The Ct values of the initial positive RT-PCR are comparable across our study.

All initially positive RT-PCR were from swabs collected with the Cepheid system. In retrospect, the Ct values of these initial RT-PCR were separated into two categories , i.e. follow-up swabs taken with the *in-house* system (white bars) and follow-up swab taken with either of all other systems (grey bars). n=26 for the *in-house* system, n=70 for all other variants. Data are depicted as mean ± SEM for the viral E gene, the viral S gene and the internal control (IC). No statistically significant differences were found. For the actual Ct values of the follow-up RT-PCRs see Figure 2.

Supplemental Figure 3

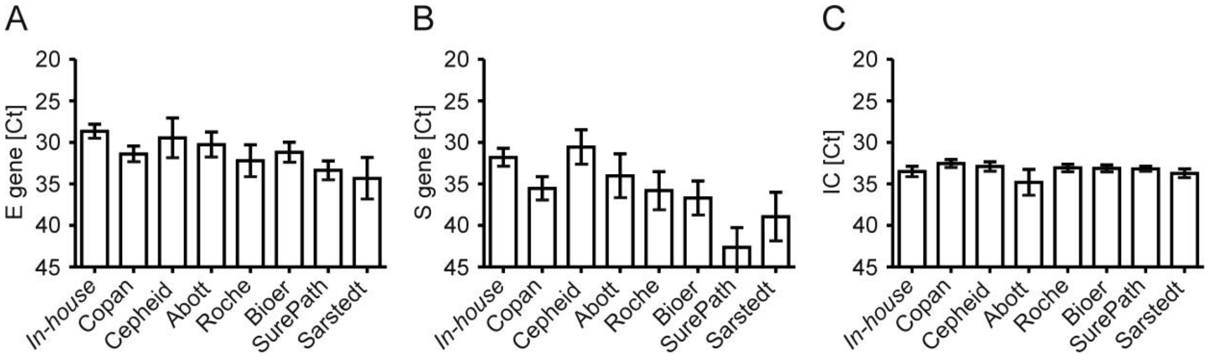

Supplemental Figure 3. Ct values of all positive RT-PCR results.

The Ct values of all positive RT-PCR results taken with either of eight swabs variants are depicted as mean ± SEM separately for the viral E gene (A), the viral S gene (B) and the internal control (C). n=26 for the *in-house* system, n=14 for the Copan system, n=10 for the Cepheid system, n=8 for the Abott system, n=11 for the Roche system, n=11 for the Bioer system, n=6 for the BD SurePath system n=10 for the Sarstedt swab. No significant difference due to the used swab type was found.

Supplemental Figure 4

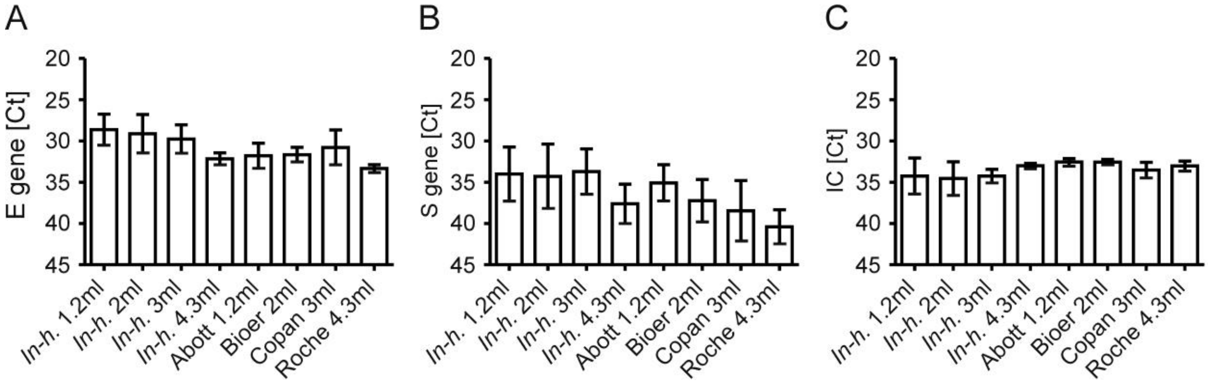

Supplemental Figure 4. No effects of the volume are detectable in the range of 1.2-4.3 ml.

To look into the effects that the volume of transport medium or preservation fluid as provided by the manufacturer may have on the RT-PCR result, we sampled in 1.2ml, 2ml, 3ml or 4.3ml of *in-house* (*In-h.*) VTM. For comparison, we chose the Abott system with 1.2ml, the Bioer system with 2ml, the Copan system with 3ml and the Roche system with 4.3ml of liquid. Ct values for the viral E gene (A), the viral S gene (B) and the internal control (IC) are depicted. n=6 independent samples. No statistically significant differences were found.

Supplemental Figure 5

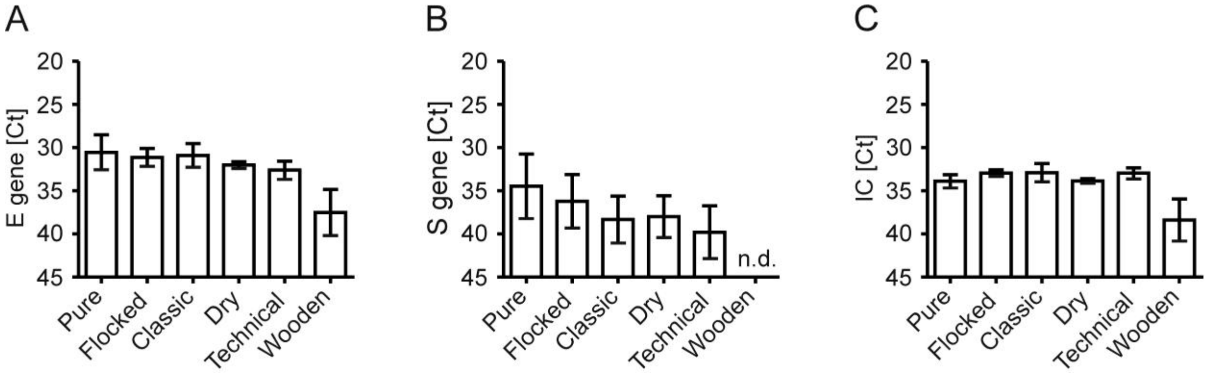

Supplemental Figure 5. Organic compounds in wooden swabs may negatively affect the sensitivity of the RT-PCR.

To assess whether the use of different swabs in combination with the *in-house* VTM tube may affect the RT-PCR result, we sampled with either of six different swabs. Ct values for the viral E gene (A), the viral S gene (B) and the internal control (IC) are depicted. n=4 independent samples. No statistically significant differences were found. n.d. for not detected.

Supplemental Figure 6

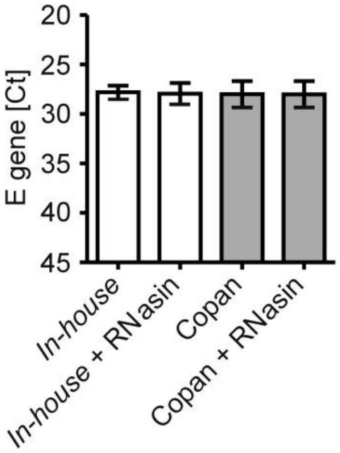

Supplemental Figure 6. RNAse inhibition may not affect the sensitivity of the RT-PCR.

To see whether an RNAse inhibitor may affect the results of RT-PCR, four samples collected with either the *in-house* VTM (white bars) or the Copan UTM (grey bars) were incubated with or without 4,000 U/ml of RNasin in the refrigerator at 2-8°C for 72 hours. No statistically significant differences in the Ct values of the viral E gene were found.
